# Supplementary material for: A UMLS-based spell checker for natural language processing in vaccine safety
Source: BMC Med Inform Decis Mak. 2007 Feb 12;7:3. doi: 10.1186/1472-6947-7-3 (PMC1805499; doi:10.1186/1472-6947-7-3)
Supplement: Additional file 13 — RAP application directory. Sets up directory for RAP (RDF application for PHP) source code [file 1472-6947-7-3-S13.gz › rap/doc/phpdoc/infModel/InfModelF.html]

xml version="1.0" encoding="iso-8859-1"?


Docs For Class InfModelF


## Class InfModelF

Description

Description |
Methods (details)

A InfModelF extends the InfModel Class, with a forward chaining algorithm.

If a new statement is added, it is enferd at once and all the entailed statements are added too. When adding or removing a statement, that produced a new inference rule, all entailed statements are discarded and the whole base model is infered again. The InfModelF is safe for loops in Ontologies, that would cause infinite loops.

History:

- 09-10-2004 : First version of this class.
- 09-15-2004 : Added Index over InRule Triggers
  to increase performance

- access: public
- author: Daniel Westphal <at d-westphal dot de mail at d-westphal dot de>
- version: V0.9.1

Located in /infModel/InfModelF.php (line 29)

```
Object
   |
   --Model
      |
      --MemModel
         |
         --InfModel
            |
            --InfModelF
```

Method Summary

Description |
Methods (details)

InfModelF
InfModelF
([string $baseURI = NULL])

void
add
(object Statement $statement)

void
addModel
(object Model &$model)

boolean
addWithoutDuplicates
(object Statement &$statement)

array
entailStatement
(object Statement &$statement)

object MemModel
getBaseMemModel
()

object MemModel
&getMemModel
()

void
load
(string $filename, [string $type = NULL])

boolean
remove
(object Statement $statement)

void
removeInfered
()

string
toString
()

Methods

Description |
Methods (details)

Constructor InfModelF (line 56)

Constructor You can supply a base\_uri

- access: public

InfModelF

InfModelF
([string $baseURI = NULL])

- string
  $baseURI

add (line 74)

Adds a new triple to the MemModel without checking if the statement is already in the MemModel.

So if you want a duplicate free MemModel use the addWithoutDuplicates() function (which is slower then add()) The statement is infered and all entailed statements are added.

- throws: PhpError
- access: public

void

add
(object Statement $statement)

- object Statement
  $statement

---

Redefinition of:

InfModel::add()
:   Adds a new triple to the Model without checking if the statement is already in the Model.

addModel (line 398)

Adds another model to this MemModel.

Duplicate statements are not removed. If you don't want duplicates, use unite(). If any statement of the model to be added to this model contains a blankNode with an identifier already existing in this model, a new blankNode is generated.

- throws: phpErrpr
- access: public

void

addModel
(object Model &$model)

- object Model
  $model

---

Redefinition of:

MemModel::addModel()
:   Adds another model to this MemModel.

addWithoutDuplicates (line 111)

Checks if a new statement is already in the MemModel and adds the statement, if it is not in the MemModel.

addWithoutDuplicates() is significantly slower then add(). Retruns TRUE if the statement is added. FALSE otherwise. The statement is infered and all entailed statements are added.

- throws: PhpError
- access: public

boolean

addWithoutDuplicates
(object Statement &$statement)

- object Statement
  $statement

---

Redefinition of:

MemModel::addWithoutDuplicates()
:   Checks if a new statement is already in the MemModel and adds the statement, if it is not in the MemModel.

entailStatement (line 172)

Entails a statement by recursively using the \_entailStatementRec method.

- return: of statements
- access: public

array

entailStatement
(object Statement &$statement)

- object Statement
  $statement

getBaseMemModel (line 308)

Create a MemModel containing only the base triples (without inferred statements) of the current InfModelF

- access: public

object MemModel

getBaseMemModel
()

getMemModel (line 290)

Create a MemModel containing all the triples (including inferred statements) of the current InfModelF

- access: public

object MemModel

&getMemModel
()

load (line 262)

Load a model from a file containing RDF, N3 or N-Triples.

This function recognizes the suffix of the filename (.n3 or .rdf) and calls a suitable parser, if no $type is given as string ("rdf" "n3" "nt"); If the model is not empty, the contents of the file is added to this DbModel.

While loading the model, the inference entailing is disabled, but new inference rules are added to increase performance.

- access: public

void

load
(string $filename, [string $type = NULL])

- string
  $filename
- string
  $type

---

Redefinition of:

Model::load()
:   Load a model from a file containing RDF, N3, N-Triples or a xhtml document containing RDF.

remove (line 333)

Removes the triple from the MemModel.

TRUE if the triple is removed. FALSE otherwise.

Checks, if it touches any statements, that added inference rules to the model

- throws: PhpError
- access: public

boolean

remove
(object Statement $statement)

- object Statement
  $statement

---

Redefinition of:

MemModel::remove()
:   Removes the triple from the MemModel.

removeInfered (line 234)

Removes all infered statements from the model but keeps the infernece rules.

- access: public

void

removeInfered
()

toString (line 279)

Short Dump of the InfModelF.

- access: public

string

toString
()

---

Redefinition of:

MemModel::toString()
:   Short Dump of the MemModel.

#### Inherited Methods


Inherited From InfModel

> InfModel::InfModel()  
> InfModel::add()  
> InfModel::findRegex()  
> InfModel::findVocabulary()  
> InfModel::getSchema()  
> InfModel::replace()

Inherited From MemModel

> MemModel::MemModel()  
> MemModel::add()  
> MemModel::addModel()  
> MemModel::addNamespace()  
> MemModel::addParsedNamespaces()  
> MemModel::addWithoutDuplicates()  
> MemModel::close()  
> MemModel::contains()  
> MemModel::containsAll()  
> MemModel::containsAny()  
> MemModel::equals()  
> MemModel::find()  
> MemModel::findAsIterator()  
> MemModel::findCount()  
> MemModel::findFirstMatchingStatement()  
> MemModel::findRegex()  
> MemModel::findVocabulary()  
> MemModel::getIndexType()  
> MemModel::getParsedNamespaces()  
> MemModel::getStatementIterator()  
> MemModel::index()  
> MemModel::intersect()  
> MemModel::isEmpty()  
> MemModel::isIndexed()  
> MemModel::iterFind()  
> MemModel::rdqlQuery()  
> MemModel::rdqlQueryAsIterator()  
> MemModel::reify()  
> MemModel::remove()  
> MemModel::removeNamespace()  
> MemModel::replace()  
> MemModel::saveAs()  
> MemModel::setBaseURI()  
> MemModel::size()  
> MemModel::subtract()  
> MemModel::toString()  
> MemModel::toStringIncludingTriples()  
> MemModel::unite()  
> MemModel::writeAsHtml()  
> MemModel::writeAsHtmlTable()  
> MemModel::writeRdfToString()

Inherited From Model

> Model::Model()  
> Model::findForward()  
> Model::getBaseURI()  
> Model::getMemModelByRDQL()  
> Model::getOntModel()  
> Model::getResmodel()  
> Model::load()  
> Model::visualize()

Inherited From Object

> Object::toString()

Documentation generated on Thu, 7 Jul 2005 13:41:42 +0200 by phpDocumentor 1.3.0RC3
